# QardioArm Blood Pressure Monitoring in a Population With Type 2 Diabetes: Validation Study

**DOI:** 10.2196/19781

**Published:** 2020-07-24

**Authors:** Victoria Mazoteras-Pardo, Ricardo Becerro-De-Bengoa-Vallejo, Marta Elena Losa-Iglesias, Eva María Martínez-Jiménez, César Calvo-Lobo, Carlos Romero-Morales, Daniel López-López, Patricia Palomo-López

**Affiliations:** 1 Facultad de Enfermería, Fisioterapia y Podología Universidad Complutense de Madrid Madrid Spain; 2 Faculty of Health Sciences Universidad Rey Juan Carlos Alcorcón Spain; 3 Departamento de Enfermería Facultad de Fisioterapia y Enfermería Universidad de Castilla la Mancha Toledo Spain; 4 Faculty of Sport Sciences Universidad Europea de Madrid Villaviciosa de Odón Spain; 5 Research, Health and Podiatry Group, Department of Health Sciences Faculty of Nursing and Podiatry Universidade da Coruña Ferrol Spain; 6 University Center of Plasencia Universidad de Extremadura Plasencia Spain

**Keywords:** blood pressure, hypertension, type 2 diabetes, mobile applications, software validation

## Abstract

**Background:**

Home blood pressure monitoring has many benefits, even more so, in populations prone to high blood pressure, such as persons with diabetes.

**Objective:**

The purpose of this research was to validate the QardioArm mobile device in a sample of individuals with noninsulin-dependent type 2 diabetes in accordance with the guidelines of the second International Protocol of the European Society of Hypertension.

**Methods:**

The sample consisted of 33 patients with type 2 diabetes. To evaluate the validity of QardioArm by comparing its data with that obtained with a digital sphygmomanometer (Omron M3 Intellisense), two nurses collected diastolic blood pressure, systolic blood pressure, and heart rate with both devices.

**Results:**

The analysis indicated that the test device QardioArm met all the validation requirements using a sample population with type 2 diabetes.

**Conclusions:**

This paper reports the first validation of QardioArm in a population of individuals with noninsulin-dependent type 2 diabetes. QardioArm for home monitoring of blood pressure and heart rate met the requirements of the second International Protocol of the European Society of Hypertension.

## Introduction

Ultimately, devices that automatically measure blood pressure have gradually evolved to replace the standard mercury sphygmomanometer, as the risk of mercury toxicity outweighed any potential benefits of its use. Today there are a wide variety of devices capable of measuring blood pressure both in and outside of clinical environments [1-7].

Among the advantages of home blood pressure monitoring are that it helps to detect white coat and masked hypertension; it is highly available at a low price; blood pressure measurements at home are taken in a more natural, relaxed, and domestic environment than when taken in the doctor’s office; patient convenience in blood pressure measurement ensures more commitment; repeated measurements can be easily taken, and it can be used over longer periods to assess the daily variability of blood pressure [8].

But logically, it is a necessary requirement that these devices have been validated by standardized protocols, such as the European Society of Hypertension-International Protocol 2 (ESH-IP2) [9], the British Hypertension Society protocol [10], or the protocol of the Association for the Advancement of Medical Instrumentation [11].

If this validation is essential in the general population, it is even more important in at-risk populations, such as patients with diabetes, where reliability and accurate blood pressure measurement are essential to avoid the disease’s progression. Type 2 diabetes is a prevalent disease and represents a very serious social health problem; it has been seen to occur to a greater extent in older adults [12,13]. The main complication of type 2 diabetes is atherosclerotic disease [14,15].

This atherosclerotic disease is the main cause of morbidity and mortality in people with diabetes, and the one that contributes the most to the direct and indirect costs of diabetes [16,17]. In addition, atherosclerosis reduces arterial elasticity, and therefore, causes arterial stiffness. This arterial wall stiffening produces a high risk of mortality [14-18]. Furthermore, the occurrence of stiffened arteries increases with diabetes [19] and hypertension; therefore, it is common that patients with diabetes are also hypertensive [18,20,21]. In fact, it is estimated that more than two-thirds of patients with type 2 diabetes are also hypertensive [22]. It has also been shown that arterial stiffness is more critical in individuals with both hypertension and type 2 diabetes than in those without hypertension [23,24]. This is a major health problem.

For all these reasons, correctly and validly measuring blood pressure in a person with diabetes is crucial. On one hand, blood pressure control has been shown to decrease the risk of diabetes-related complications such as microvascular and microvascular pathology [16,22-25]. On the other hand, the increase in arterial stiffness can affect the accuracy of automatic blood pressure measurements that are essential for diagnosis and administration [26,27]. And, more importantly, measuring blood pressure accurately facilitates the establishment of a threshold above which antihypertensive treatment may be recommended, especially in patients with type 2 diabetes who are very likely to develop complications such as peripheral arterial disease, stroke, heart attack, sudden death from heart failure, or renal pathologies, if not treated [28,29].

In that regard, it has been proven that blood pressure control and the prevention of morbidity and related mortality could be improved in persons with diabetes [3,8,16]. However, knowledge, treatment, and control of hypertension are persistently low worldwide [30,31].

One of the great challenges is to avoid therapeutic inertia, as this would result in an unacceptable burden in terms of human lives, sequelae, and socioeconomic costs [16,32].

Therefore, we consider it highly relevant to evaluate the validity of automatic blood pressure measurements in persons with diabetes because of the high prevalence of hypertension in this specific population and its significant morbidity and mortality rate.

The main goal of this study was to test the validity of the measurements of the QardioArm blood pressure monitoring device in individuals with noninsulin-dependent type 2 diabetes persons, in accordance with ESH-IP2 [9]. We hypothesized that the QardioArm for home blood pressure monitoring measurements of blood pressure and heart rate in individuals with noninsulin-dependent type 2 diabetes would be equivalent to those from a gold standard device within the requirements of ESH-IP2.

## Methods

### Study Design

We performed an observational concordance study to validate the QardioArm device for measurement of heart rate, diastolic blood pressure, and systolic blood pressure, in individuals with noninsulin-dependent type 2 diabetes according to ESH-IP2 [9] and STROBE (Strengthening the Reporting of Observational Studies in Epidemiology) criteria [33]. The study was conducted between September 2019 to January 2020.

### Ethics

This research study received ethical approval (number 173/2019). This study respected the Helsinki Declaration [34]. All participants signed an informed consent form prior to being included in the study.

### Devices

#### Omron M3 Intellisense

The Omron M3 Intellisense (Omron Healthcare Co Ltd) was used as the gold standard instead of a mercury sphygmomanometer, in this study, because it has been validated in the general population according to ESH-IP2 [35].

The Omron M3 Intellisense device records heart rate in the range of 40 to 180 bpm and brachial blood pressure in the range of 0 to 299 mmHg using the oscillometric method; systolic blood pressure, diastolic blood pressure, and heart rate are shown on screen. The inflation system does not require pressure presetting or reinflation because of its technology, and deflation is automatically released by a pressure valve. It weighs approximately 340 g without batteries. The standard cuff fits arm circumferences ranging from 22 to 32 cm; it is also available in a large cuff to fit arm circumferences from 32 to 42 cm.

#### QardioArm

The QardioArm (Atten Electronic Co) is an automatic home blood pressure measurement monitoring device. QardioArm is a blood pressure measurement system intended to assess heart rate, diastolic blood pressure, and systolic blood pressure in an adult population, for pulse rates in the range of 40 to 200 bpm and blood pressure in the range of 40 to 250 mmHg.

This device utilizes an inflatable cuff that is wrapped around the upper arm, with circumferences ranging from 22 to 37 cm. The weight of unit is 310 g with batteries and its dimensions, when closed, are 140 mm×68 mm×38 mm. There is a freely downloadable app on the company’s website, Google Play, or the Apple App Store. A smart device with Android 4.4 (KitKat) or later, or Bluetooth 4.0 and iOS 7.0 or later, is required, being compatible with iPhone, iPod, iPad, Apple Watch, tablets, and smartphones. QardioArm also provides graphics to facilitate visual data interpretation on screen. This app may be configured by warnings and reminders, also measurements and progress may be shared in real time with clinicians and other users.

### Patients and Recruitment

All patients were recruited from Fresenius Medical facilities in Plasencia-Extremadura, Spain. The inclusion criteria were age greater than 25 years, gender (at least 10 men and 10 women), and recruitment blood pressure requirements according to the guidelines [9]: 33 participants diagnosed with noninsulin-dependent type 2 diabetes were included.

The exclusion criteria were having an arrhythmia or using insulin as treatment at the time of the study.

### Study Protocol

Two nurses with experience in blood pressure measurement performed all assessments. The assessment room was a comfortable temperature without noise, distractions, or any factors that could have influenced the measurements [9].

Birth date, gender, weight, and height of each participant were recorded, and the arm circumference was measured in order to ensure an adequate cuff size. Body mass index was calculated using the Quetelet index.

The same assessment room was used for all participants. Each participant was seated in the assessment room, and the measurements were assessed after a period of 10 to 15 minutes of rest. Measurements by both units were taken on the left arm supported at the heart level, and a total of 9 consecutive measurements (systolic blood pressure, diastolic blood pressure and heart rate) were carried out alternating the Omron M3 Intellisense and the QardioArm in the following order: blood pressure A—entry blood pressure using the Omron M3 as standard device; blood pressure B—entry blood pressure using the Omron M3 as test device; blood pressure 1 by Omron M3; blood pressure 2 by QardioArm; blood pressure 3 by Omron M3; blood pressure 4 by QardioArm; blood pressure 5 by Omron M3; blood pressure 6 by QardioArm; and blood pressure 7 by Omron M3.

During measurement, the patient remained quiet and calm, sitting placing the back straight maintaining the feet over the floor in parallel position without crossing their legs as well as resting the arm over a flat surface, with the hand palm upwards and the elbow in a slightly flexed position in order to place his fist at the height of its heart. The interval between one measurement and the next was 30 or 60 seconds [9].

### Data Analysis

Results are described as mean and standard deviation with range. Sociodemographic variables were examined using the Kolmogorov–Smirnov test to assess normality, and data were considered normally distribution if *P*>.05. Two-tailed independent *t* tests or Mann–Whitney *U* tests were used for parametric or nonparametric variables, respectively.

Device accuracy, following the ESH-IP2 [9], is based on a comparison between the device and reference measurements. Part 1 of the protocol refers to the number of differences within the required ranges for individual measurements (99 measurements), and part 2 of the protocol refers to the number of differences within the required ranges for each individual (n=33).

For each patient, systolic blood pressure measurements obtained from the QardioArm device (blood pressure measurements 2, 4, and 6) were compared with the systolic blood pressure measurements from the Omron device (blood pressure measurements 1, 3, and 5, respectively, or with blood pressure measurements 3, 5, and 7, respectively); the comparisons which were favorable to the device were utilized. The same procedure was followed with diastolic blood pressure and heart rate measurements.

Differences were separately classified for diastolic blood pressure and systolic blood pressure, depending on whether the difference was within 5, 10, or 15 mmHg [9] as well as for heart rate depending on whether the difference was within 3, 5, or 8 bpm.

Results were analyzed and detailed according to ESH-IP2 requirements in order to determine if the device passed the validation protocol. Accuracy was determined by the number differences in these ranges for both individual measurements (part 1) and individuals (part 2). To pass, a device must meet the minimum pass requirements.

Furthermore, Bland-Altman plots were utilized to quantify agreement between such systolic blood pressure, diastolic blood pressure, and heart rate by constructing limits of agreement (a graphical method in which the differences between both devices are used to compare two measurements of the same variable).

All analyses were carried out with SPSS statistical software (version 19.0; IBM Corp). In all analyses, the threshold for statistical significance was *P*=.05 with a 95% confidence interval.

## Results

### Participants

All the sociodemographic variables showed a normal distribution (*P*>.05, Table 1). A sample of 37 participants diagnosed with noninsulin-dependent type 2 diabetes were recruited; 4 were excluded due to device failure (n=2), arrhythmias (n=1), and cuff size unavailability (n=1), therefore, there were 33 participants (17 men and 16 women) who met the ESH-IP2 inclusion criteria. Table 1 shows participant demographic characteristics.

**Table 1:** Demographic characteristics and descriptive data of the participants.

Participant characteristics (N=33)		Value, mean (SD)	Value, range^a^	*P* value
**Age (years)**		65.9 (9.8)	39.0-85.0	.73
	Men (n=17)	66.4 (12.3)	39.0-85.0	
	Women (n=16)	72.6 (6.7)	65.0-84.0	
**Weight (kg)**		74.8 (12.6)	48.0-101.0	.42
	Men (n=17)	76.5 (12.5)	48.0-100.0	
	Women (n=16)	72.9 (12.9)	57.0-101.0	
**Height (cm)**		164.9 (7.3)	147.0-180.0	.17
	Men (n=17)	166.6 (5.3)	160.0-180.0	
	Women (n=16)	163.1 (8.8)	147.0-175.0	
**BMI (kg/m^2^)**		27.6 (5.2)	18.1-43.7	.90
	Men (n=17)	27.5 (3.7)	18.1-34.1	
	Women (n=16)	27.7 (6.5)	20.3-43.7	
**Arm circumference (mm)**		286.4 (32.7)	220.0-360.0	.19
	Men (n=17)	279.1 (26.8)	220.0-320.0	
	Women (n=16)	294.1 (37.3)	243.0-360.0	

^a^minimum to maximum

### Blood Pressure Outcome Measurements

Parts 1 and 2 of the validation according to the ESH-IP2 for the QardioArm blood pressure are presented in Table 2; the number of differences between the device and reference for systolic blood pressure and diastolic blood pressure classified within 5, 10, or 15 mmHg are detailed.

Mean differences between the QardioArm and Omron M3 were 3.37 (SD 3.19) mmHg for diastolic blood pressure and 3.15 (SD 4.67) mmHg for systolic blood pressure.

A total of 87/99 differences (89%) for systolic blood pressure and a total of 88/99 differences (89%) for diastolic blood pressure showed an absolute difference within 5 mmHg (ESH-IP2 criteria: at least 65 for diastolic blood pressure and at least 73 for systolic blood pressure). Furthermore, a total of 95/99 comparisons (96%) for systolic blood pressure and a total of 96/99 (97%) for diastolic blood pressure showed an absolute difference within 10 mmHg (ESH-IP2 criteria: at least 81 for diastolic blood pressure and at least 87 for systolic blood pressure). A total of 98/99 differences (99%) for systolic blood pressure and a total of 99/99 differences (100%) for diastolic blood pressure exhibited an absolute difference within 15 mmHg (ESH-IP2 criteria: at least 93 for diastolic blood pressure and at least 96 for systolic blood pressure). Part 1 of the validation was successfully completed since 2 or more of the 3 absolute difference ranges (within 5, 10, or 15 mmHg) for systolic blood pressure met the minimum requirements, and 3 out of 3 absolute difference ranges for diastolic blood pressure met the minimum requirements.

A total of 32/33 patients (97%) for systolic blood pressure and a total of 29/33 patients (88%) for diastolic blood pressure showed a minimum of 2 out of 3 differences within 5 mmHg (ESH-IP2 criteria: at least 24 out of 33 patients for systolic blood pressure and at least 24 out of 33 patients for diastolic blood pressure). Nevertheless, 1/33 patients (3%) had 3 differences outside 5 mmHg for systolic blood pressure, though 0/33 patients (0%) had 3 differences outside 5 mmHg for diastolic blood pressure (ESH-IP2 criteria: a maximum of 3 patients for diastolic blood pressure and systolic blood pressure according to the ESH-IP2 criteria. Part 2 of the device validation was successfully completed; therefore, part 3 of the QardioArm device validation was passed, since parts 1 and 2 were both passed for diastolic blood pressure and systolic blood pressure.

**Table 2:** Number of measurement differences in each range.

Measurement type and range			ESH-IP2 requirement, n				Achieved (N=99), n		Difference (mmHg), mean (SD)	
				For 2 of 3 ranges		For all 3 ranges				
**Part 1**										
	**Systolic blood pressure**									3.37 (3.19)
		≤5 mmHg		73		65		87		
		≤10 mmHg		87		81		95		
		≤15 mmHg		96		93		98		
	**Diastolic blood pressure**									3.15 (SD 4.67)
		≤5 mmHg		73		65		88		
		≤10 mmHg		87		81		96		
		≤15 mmHg		96		93		99		
	**Heart rate**									1.65 (2.91)
		≤3 bpm		73		65		91		
		≤5 bpm		87		81		96		
		≤8 bpm		96		93		99		

### Heart Rate Outcome Measurements

Parts 1 and 2 of the validation according to the ESH-IP2 for heart rate are also shown in Table 2 and Table 3; the number of differences between QardioArm and the standard device, Omron M3, within 3, 5, and 8 bpm are detailed. The mean difference between the tested device and standard was 1.65 (SD 2.91) bpm.

A total of 91/99 differences (92%) showed an absolute difference within 3 bpm, a total of 96/99 comparisons (97%) showed an absolute difference within 5 bpm, and a total of 99/99 differences (100%) showed an absolute difference within 8 bpm. Thus, part 1 of the device validation was successfully completed for the heart rate.

**Table 3:** Within-participant measurement differences.

Measurement type and range			2 of 3 measurements			0 of 3 measurements	
			ESH-IP2 requirement, n		Achieved (N=33), n	ESH-IP2 requirement, n	Achieved (N=33), n
**Part 2**							
	**Systolic blood pressure**						
		≤5 mmHg	≥24		32	≤3	1
	**Diastolic blood pressure**						
		≤5 mmHg	≥24		29	≤3	0
	**Heart rate**						
		≤3 bpm	≥24		31	≤3	1

A total of 31/33 participants (94%) had a minimum of 2 of 3 comparisons within 3 bpm difference for heart rate. Nevertheless, a total of 1/33 participants (3%) had 3 differences greater than 3 bpm; therefore, part 2 of the device validation was successfully completed for heart rate, and consequently, part 3 of the QardioArm device validation was passed.

Indeed, the QardioArm device met the validation criteria of the ESH-IP2 for heart rate, systolic blood pressure, and diastolic blood pressure for this sample of 33 individuals with noninsulin-dependent type 2 diabetes.

**Figure 1:**
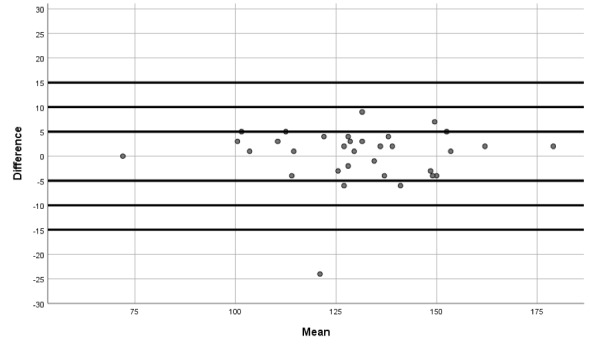
Bland-Altman plot showing differences between QardioArm and Omron M3 measurements for systolic blood pressure.

**Figure 2:**
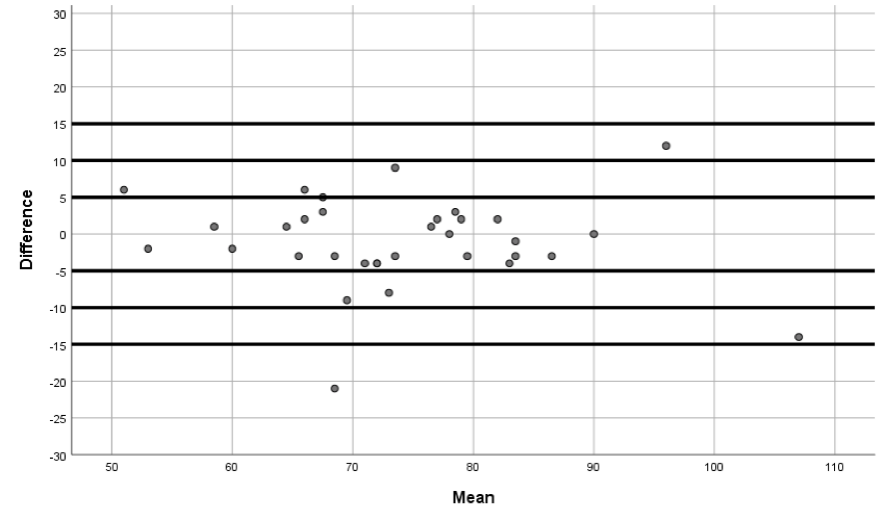
Bland-Altman plot showing differences between QardioArm and Omron M3 measurements for diastolic blood pressure.

**Figure 3:**
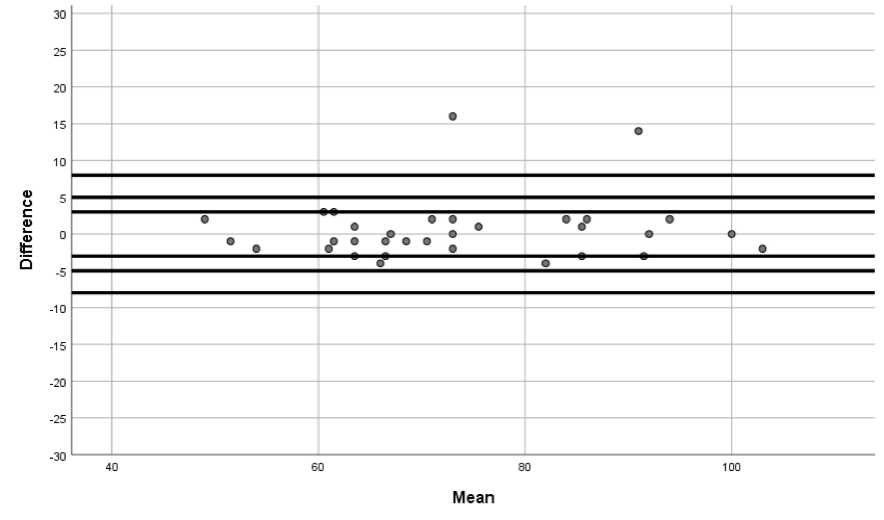
Bland-Altman plot showing differences between QardioArm and Omron M3 measurements for heart rate.

The Bland-Altman plots show the differences between QardioArm and Omron M3 measurements for systolic blood pressure (Figure 1), diastolic blood pressure (Figure 2), and heart rate (Figure 3).

## Discussion

As far as we know, this is the first study to validate an upper arm oscillometric wireless device connected to an app to measure heart rate and blood pressure in a population with noninsulin-dependent type 2 diabetes. QardioArm was validated in 2017 [36,37] in the general population. Furthermore, QardioArm has also been validated in a population of obese individuals in 2018 [38], and recently, in a population of individuals with chronic kidney disease [39].

The results of this study have shown that QardioArm device passed both parts of the validation [9] in patients diagnosed with noninsulin-dependent type 2 diabetes as hypothesized. This validation has been carried out following the ESH-IP2 guidelines, although a validated noninvasive oscillometric upper arm device was used as a reference instead a mercury sphygmomanometer [3-6]. Our findings, however, should not be extrapolated to other populations with other diseases.

We have already seen the enormous advantages of home blood pressure monitoring, especially in specific populations prone to hypertension, such as persons with diabetes. Chronic diseases such as diabetes are one of the most common pathologies and have significant physical, psychological, social, and economic impacts [3,8,16,17,19-25,28,29,32]. mHealth and apps that assess blood pressure are progressively gaining a fundamental role in the management of hypertension with the potential to improve the quality of managed care by offering additional advantages, especially when it comes to blood pressure self-measurement. Advantages could include assisting in lowering blood pressure [3,40,41]; enabling individuals to have 24-hour access to detailed and personalized blood pressure information [40,42]; improving adherence to treatment [3,43]; providing information to facilitate diagnostic and therapeutic decisions [40,41,43,44]; improving patient knowledge of blood pressure, lifestyle, and risk factors that may accompany hypertension [40,42,43]; more effectively preventing cardiovascular complications [43]; or health cost savings by avoiding unnecessary hypertensive treatment and reducing the number of visits to the clinic [3,42].

Nevertheless, we found very few studies analyzing the validity of automatic blood pressure monitors in a population with noninsulin-dependent type 2 diabetes, [37,45-48], and furthermore, most of these validations did not follow specific protocols such as ESH-IP2 [9-11].

Only one study by Chahine et al [37] was found that followed the same recommendations as those of the ESH-IP2 [9]. They validated Omron M6 IT Comfort using a mercury sphygmomanometer as the standard. If we compare our results with their results [37], the number of differences for each category (5, 10, and 15 mmHg) for the systolic blood pressure and the diastolic blood pressure were similar in the two validations, and parts 1 and 2 were passed in both. QardioArm achieved better results in part 1 of the protocol, especially for diastolic blood pressure with a number of higher differences in the 3 categories. Within part 2, QardioArm and Omron M6 differed in 3 individuals for the first criterion (2 of 3 comparisons within 5 mmHg difference) for both systolic and diastolic pressure, in favor of QardioArm. The results of the second criterion (3 differences outside 5 mmHg) were almost identical for both devices. On the other hand, the data obtained for heart rate cannot be compared because Chahine and company [37] did not assess this variable.

Another important fact is that measurements of people with type 2 diabetes involve stiffer arteries; however, in our study this was not assessed, since it was not the main aim of this research.

Finally, for future studies, consecutive sampling bias should be considered; a simple random sampling process might be more appropriate.

Moreover, we consider that further validations are needed for devices that measure blood pressure in patients with noninsulin-dependent type 2 diabetes. In fact, a future line of research could be to specifically investigate the validation of this QardioArm device in patients with type 2 diabetes who have microarteriopathy as diabetic retinopathy, patients with type 1 diabetes as well as other populations with specific diseases such as pregnant women.

QardioArm for home blood pressure monitoring showed validated measures of blood pressure and heart rate in individuals with noninsulin-dependent type 2 diabetes meeting the requirements of ESH-IP2. The findings of the study may be relevant since it is the first validation showing that a device linked to an app to measure blood pressure and heart rate met the requirements of the ESH-IP2 in noninsulin-dependent type 2 diabetes population.

## Abbreviations

ESH-IP2: second International Protocol of the European Society of Hypertension
